# Statements for conducting high-resolution manometry during the COVID-19 pandemic

**DOI:** 10.3164/jcbn.20-97

**Published:** 2020-08-22

**Authors:** Hideki Mori, Jan Tack, Hidekazu Suzuki

**Affiliations:** 1 Department of Clinical and Experimental Medicine, Translational Research Center for Gastrointestinal Diseases (TARGID), University of Leuven, Leuven 3000, Belgium; 2 Division of Gastroenterology and Hepatology, Department of Internal Medicine, Tokai University School of Medicine, 143 Shimokasuya, Isehara, Kanagawa 259-1193, Japan

**Keywords:** SARS-CoV-2, COVID-19, high-resolution manometry, motility disorders of the gastrointestinal tract, infection prevention

## Abstract

To ensure the safety of medical personnel is important during the new coronavirus infectious disease (COVID-19) pandemic. Although high-resolution manometry (HRM) is an essential device for diagnosis of functional gastrointestinal disorders, it contains risks of droplet infection, contact infection and aerosol-borne infection. Screening tests such as PCR, serology test to detect COVID-19 antibodies, and CT scan should be considered as well as body temperature check and anamnestic risk assessment. Moreover, the provision of protective equipment such as a mask with face shield (or goggles + mask), gloves, cap or hairnet, and a long-sleeved gown would be necessary to reduce the risk of COVID-19.

## Introduction

In the pandemic phase following the spread of the new coronavirus infectious disease (COVID-19), in Japan, as in US or EU, emergency declarations have been issued nationwide, and the Japanese Society of Gastroenterology (JSGE) and Japan Gastroenterological Endoscopy Society (JGES) recommend postponement of non-emergency endoscopy from the perspective of protecting endoscopists and patients. Similar policies should be followed for high-resolution manometry (HRM) that is considered to have the same risk of infection as endoscopy. However, in the early recovery phase, there is a demand for recommending HRM, in consideration of the safety of medical staff and patients.^(1–4)^ Based on the available evidence, we are proposing the following statements regarding HRM in consideration of the current situation of COVID-19.

## COVID-19 Infection Route and HRM

The infection routes of coronavirus are basically droplet infection and contact infection, the same applies to SARS-CoV-2.^(5)^ Positioning of HRM catheters for gastrointestinal motility tests may also elicit cough or sneezing in patients and hence carries a concern for aerosol-borne infection of health care workers.^(6,7)^ A high frequency of transmission of the virus by aerosol is likely when subjects are exposed to a high concentration of contaminated aerosol in a closed space such as an examination room for a certain period of time. In addition, HRM catheters can become contaminated with body fluids, so care must be taken when these are handled to avoid infection.

## The Screening for HRM Avoiding Risks of COVID-19

Motility disorders of the gastrointestinal tract are generally chronic, and rarely can be considered urgent. We therefore suggest to postpone HRM tests in cases with confirmed or clinically suspected COVID-19, until the (suspected) infection has subsided (Table 1).^(8–12)^ If subjects have a history of close contact with COVID-19 patients or have a travel history to an outbreak area within the past 2 weeks, HRM tests should also be postponed. Since there is a risk that also asymptomatic COVID-19 patients can spread the infection, it is recommended to perform HRM tests using the strict criteria listed in Table 2 in the early recovery phase of the COVID-19 epidemic situation.^(13)^ In other words, it is necessary to prove, either by the antibody titer (if a reliable assay is available) that the subjects has already been infected or to confirm that there is no acute infection by a negative PCR test for COVID-19 on a nasal or pharyngeal swab.^(14)^ As a back-up option, thoracic CT scan has also been advocated as a method for identifying acutely infected patients.^(3,15)^

## Check and Environment in the Laboratory

Patients should wear a surgical mask for their arrival at and stay in the hospital. On the day of HRM test, it is recommended that a screening interview is held to establish that the subject did not develop any novel symptoms suggestive for COVID-19, that there was no contact with an infected subject, and that the subject’s temperature is measured before entering the examination room. In case of new onset symptoms, exposure or a temperature rise, the examiner should consider postponement of the HRM. In addition, in order to prevent droplet infection and contact infection in the examination room, the examiner should consider arranging an environment where all subjects (including attendants) can keep a safe distance.^(15)^ In a room with windows, if possible, open the windows on opposite or different sides simultaneously to encourage ventilation. During transnasal positioning and advancing of the HRM catheter, the patient should continue to wear a surgical mask over the mouth.

## Protection for the Staff Performing HRM Tests

Even with strict screening, false negatives can occur,^(16)^ so as a general rule examiners must take standard precautions to prevent infection from COVID-19 when performing HRM tests. It is recommended to practice the maximum possible infection prevention according to the availability of resources such as personal protective equipment at each facility. Personnel should wear a mask with face shield (or goggles + mask), gloves, cap or hairnet, and a long-sleeved gown. Protective equipment should be replaced for each patient, and the examiner should thoroughly wash the hands after HRM tests.

In addition, when cleaning after HRM tests, disinfection of catheters will be performed as usual with sufficient protective measures. Enveloped viruses such as coronaviruses are the least resistant to inactivation by disinfection.^(17)^ In order to prevent contact infection, it is necessary to thoroughly disinfect not only the catheters but also the peripherals such as the used PC, table and bed.^(3)^ When available, the use of a disposable probe cover sheath or condom is recommended to reduce the level of microbial exposure. It is also recommended that all catheters and probes are cleaned and disinfected after each procedure, with a chlorine dioxide based or comparable disinfectant.

## Figures and Tables

**Table 1:** Confirmed and suspected COVID-19 cases and high risk state of COVID-19

Confirmed COVID-19 cases
Subjects who tested positive for COVID-19
Suspected COVID-19 cases
Common cold symptoms (runny nose, sneezing, fatigue, cough)
A body temperature of 37.5°C or higher
Strong fatigue and stuffiness
Dysgeusia and dysosmia without apparent cause
Digestive symptoms such as diarrhea lasting 4–5 days without apparent cause
High risk state of COVID-19
History of close contact with COVID-19 patients within 2 weeks
Travel history to an outbreak area within 2 weeks

**Table 2:** Screening options before HRM test

Confirmed negative by PCR test the day before the test
Antibody test for IgG positive and IgM negative
CT scan of the lungs negative
